# The Cold Atmospheric Plasma Inhibits Cancer Proliferation Through Reducing Glutathione Synthesis

**DOI:** 10.3390/molecules30132808

**Published:** 2025-06-30

**Authors:** Qiyu Yang, Wei Zhao, Lingling Yang, Yongqin Fan, Changsheng Shao, Tao Wang, Fengqiu Zhang

**Affiliations:** 1 School of Physics and Laboratory of Zhongyuan Light, Zhengzhou University, Zhengzhou 450001, China; yangqiyu0711@163.com (Q.Y.); 18337395772@163.com (W.Z.); 2 Henan Key Laboratory of Ion-Beam Green Agriculture Bioengineering, Zhengzhou University, Zhengzhou 450001, China; yanglingling00123@163.com (L.Y.); fanyongqin_1@163.com (Y.F.); 3 CAS Key Laboratory of High Magnetic Field and Ion Beam Physical Biology, Hefei Institutes of Physical Sciences, Chinese Academy of Sciences, Hefei 230031, China; shaochangsheng@hmfl.ac.cn; 4 College of Nursing and Health, Zhengzhou University, Zhengzhou 450001, China; 5 The Kids Research Institute Australia, The University of Western Australia, Nedlands, WA 6872, Australia

**Keywords:** cancer, cold atmospheric plasma, esophageal squamous carcinoma, glutamine metabolism, glutathione

## Abstract

(1) Objective: Cold atmospheric plasma (CAP) is a safe and effective alternative to radiotherapy for cancer treatment. Its anticancer effects are attributed to increased intracellular reactive oxygen species (ROS). Glutathione, a key antioxidant derived from glutamine, is critical for cell proliferation. This study investigated whether CAP-induced ROS elevation results from reduced glutamine–glutathione conversion and elucidates the underlying mechanisms. (2) Methods: Using esophageal squamous carcinoma cell models (Ec9706 and Eca109), we analyzed CAP’s effects on key enzymes in glutamine metabolism (Glutaminase 1 and γ-glutamylcysteine ligase) and proliferation-related genes (e.g., Retinoblastoma and Nuclear respiratory factor 2). Transcriptome analysis further explored molecular pathways involved in CAP-mediated anticancer effects. (3) Results: CAP reduced Glutaminase 1 and γ-glutamylcysteine ligase expression, leading to lower intracellular glutathione, higher ROS activity, and enhanced apoptosis. Transcriptome data confirmed CAP’s role in oxidation-reduction reactions and glutamine metabolism. (4) Conclusions: This study provides the first mechanistic insights into CAP’s anticancer effects by targeting glutamine metabolism. While based on in vitro assays, these findings guide the development of novel CAP therapies for currently incurable cancers.

## 1. Introduction

Cold atmospheric plasma (CAP) is the fourth state of substances besides liquid, solid, and gas [1]. It is composed of a mix of ionized and non-ionized particles, including electrons, ions, neutral atoms in ground/excited states, and free radicals (e.g., reactive oxygen species and reactive nitrogen species) [2]. These endow CAP with unique features for broad biomedical applications [3]. For instance, after interacting with cells, CAP causes instantaneous openings on the cell membrane (typically exceeding microseconds), which enables CAP to have great potential in in vitro/in vivo gene transfection [4]; importantly, when cancer cells are stimulated, these spontaneous opening on cell membrane provide an opportunity for the delivery of free radicals contained in CAP for cancer treatment [5,6]. Indeed, in a way similar to radiotherapy, CAP has shown great promise in the treatment of multiple types of cancers, such as melanoma [7], skin cancer [8], colon cancer [9], breast cancer [4], neuroblastoma [10], ovarian cancer [11], and glioblastoma [12], via either monotherapy or a combination with other treatments. Notably, compared with traditional radiotherapy, CAP is of great advantage in terms of both efficacy and safety, as shown in Table 1. 

Recently, we found that these CAP-mediated anticancer effects involve the inhibition of glutathione [18]. As glutathione is a well-described protective antioxidant in our bodies, via inhibiting the cellular level of glutathione, the free radicals released from CAP treatment could impose massive damage on cancer cells [19,20,21]. However, the detailed mechanisms of how CAP affects glutathione production are yet to be elucidated. As the main source of glutathione, glutamine is one of the most abundant amino acids in the tumor microenvironment [22,23]. The metabolism of glutamine is, therefore, very likely to affect glutathione synthesis. While the production of glutamine is driven by an irreversible reaction catalyzed by glutaminase (GLS1) and γ-glutamylcysteine ligase [24], its degradation involves several aminotransferases, such as glutamate dehydrogenase [25,26]; affecting these factors could affect the glutamine–glutathione transition. Here, by employing two established CAP-stimulating esophageal cancer models, for the first time, the influence of these pathways in glutathione synthesis after CAP stimulation was systematically explored. This study not only improves our understanding of how CAP kills cancer cells from the molecular level but also provides knowledge to further improve the efficacy of CAP-mediated cancer treatment.

## 2. Result

### 2.1. CAP Inhibits Cell Proliferation by Enhancing Intracellular ROS Level

**Figure 1:**
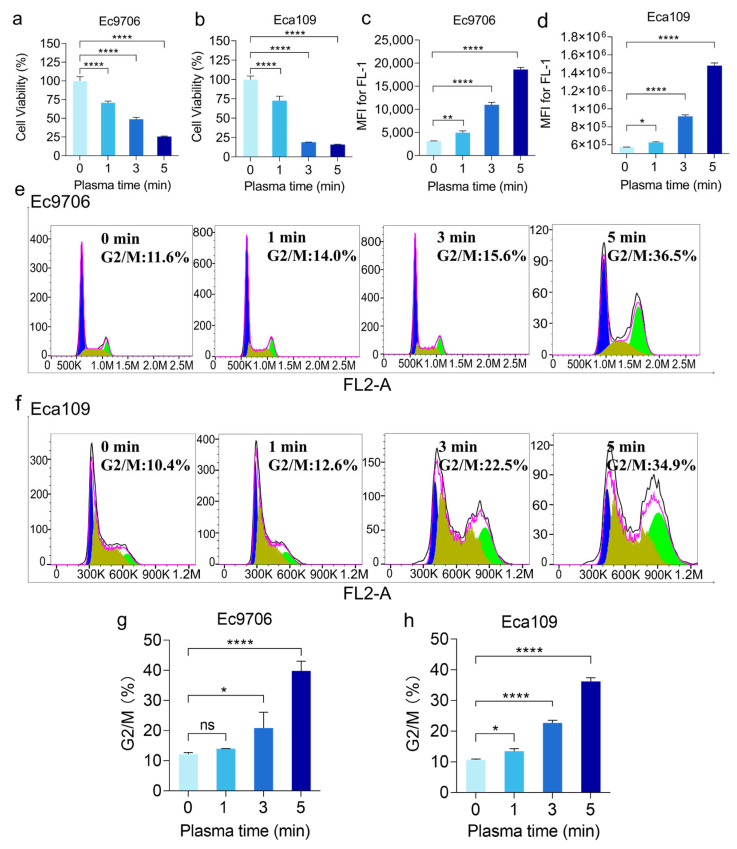
CAP treatment inhibits esophageal cancer cell proliferation through ROS-induced cell cycle blocked at G2/M. (**a**,**b**) CAP inhibits the growth of both Ec9706 and Eca109 cells. (**c**,**d**) ROS level increase was observed after CAP treatment. (**e**,**f**) Results of the cell cycle analysis of Ec9706 and Eca109 cells through flow cytometry. (**g**,**h**) The proportion of cells in the G2/M phase increased in both Ec9706 and Eca109 cells as the CAP treatment time progressed. (* representing *p* < 0.05; ** representative *p* < 0.01; **** representative *p* < 0.0001; ns represents *p* > 0.05).

We first assessed the cytotoxicity of CAP on two esophageal squamous carcinoma cell lines, Ec9706 and Eca109, using the MTT assay [27]. As shown in Figure 1a,b, CAP could significantly inhibit cell proliferation in a time-dependent manner in both cell lines. For example, after 1, 3, and 5 min of CAP treatment, Ec9706 cells showed a significant decrease in vitality compared with untreated cells (0 min), with a fourfold decrease after 5 min of CAP treatment (Figure 1a). A similar phenomenon was observed in Eca109 cells (Figure 1b). This indicates that CAP can significantly inhibit the proliferation of esophageal cancer cells.

Because CAP treatment generates a large amount of ROS, which has been suggested to be crucial for CAP-induced cell death, we analyzed the intracellular ROS levels using 2′, 7′-Dichlorofluorescin diacetate (DCFH-DA). As shown in Figure 1, the DCF fluorescence of cells significantly increased with the increase of CAP doses (Figure 1c,d), indicating an increased intracellular ROS level after CAP treatment. 

Then, we further investigated this CAP-mediated cell death by analyzing cell cycles. Indeed, we found that cell proliferation was inhibited after CAP treatments. As shown in Figure 1e–h, about 36.5% of Ec9706 cells treated with 5 min CAP were blocked in the G2/M phase, which is significantly higher than that of the untreated cells (11.6%). Similarly, approximately 34.9% of Eca109 cells treated with CAP for 5 min were arrested in the G2/M phase compared with the 10.4% G2/M phase of the 0 min treatment. As G2/M is the critical period of cancer cell mitosis [28], these results indicate that CAP treatment inhibits cancer cell division by enhancing the level of ROS.

### 2.2. CAP Kills Cancer Cells Through Inducing Both Apoptosis and Necrosis

**Figure 2:**
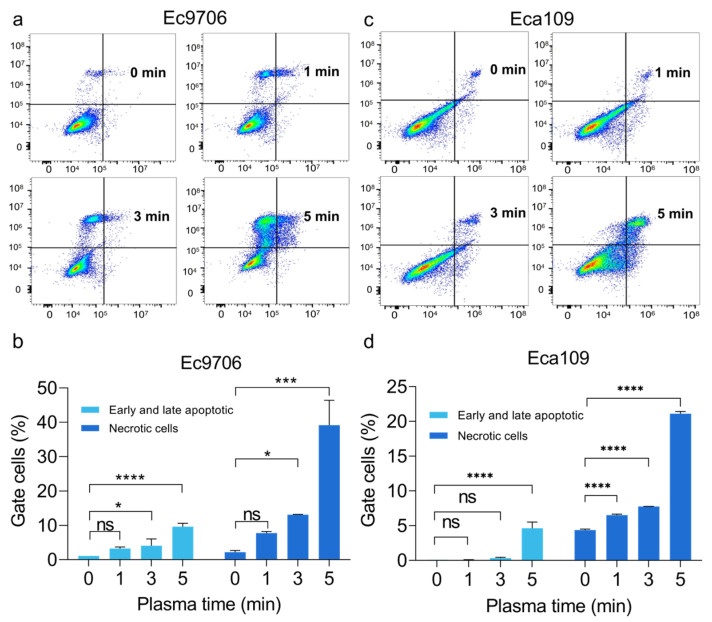
CAP treatment induces apoptosis in both Ec9706 and Eca109 cells. (**a**,**c**) The apoptosis analysis of Ec9706 cells and Eca109 cells was shown by flow cytometry. (**b**,**d**) The proportion of apoptosis and necrosis of both types of cells increased as the treatment time lengthened. (* representing *p* < 0.05; *** Representing *p* < 0.001; **** representative *p* < 0.0001; ns represents *p* > 0.05).

Then, we studied how CAP affected the fate of cells in more detail by further analyzing the levels of apoptosis and necrosis of esophageal squamous carcinoma cells. As illustrated in Figure 2, compared with the control group, both Ec9706 and Eca109 cells showed a significant rise in apoptosis and necrosis after treatments of 1 min, 3 min, and 5 min. For instance, after 5 min treatment, Ec9706 cells showed a significant increase in apoptosis from 1.12% to 9.64% compared with 0 min treatment, and a significant increase in cell necrosis was recorded from 2.22% to 39.15% for the untreated group and the 5 min treated groups. Similar apoptosis and necrosis results were also observed with Eca109 cells. These results suggested that the potent anticancer effects of CAP involved both apoptosis and necrosis.

### 2.3. CAP Treatment Results in the Damage of Key Intracellular Macromolecules: DNA, Protein and Lipid

**Figure 3:**
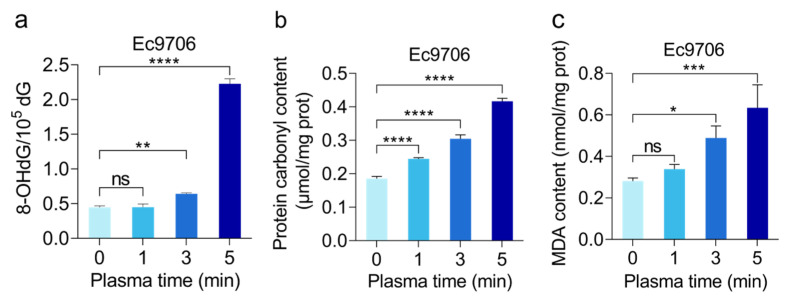
The level of oxidative damage in esophageal cancer cells after CAP treatment. The relative content of DNA damage marker 8-OHdG (**a**), protein carbonyl content (**b**), and MDA content (**c**) in Ec9706 cells all increased as the treatment time lengthened. (* representing *p* < 0.05; ** representative *p* < 0.01; *** Representing *p* < 0.001; **** representative *p* < 0.0001; ns represents *p* > 0.05).

Based on the above results, we further explored the ROS-induced anticancer effects from molecular levels. As is known, 8-Hydroxydeoxyguanosine (8-OHdG) is a major biomarker of DNA oxidative damage [29], which can be produced by reactive oxygen species such as hydroxyl radicals, singlet oxygen, etc., attacking the 8th carbon atom of the guanine bases in DNA molecules [30,31]. To test if CAP treatment can affect DNA structure, we analyzed the content of 8-OHdG in Ec9706 cells after CAP treatment using HPLC-MS. We found that the intracellular 8-OHdG level could be dramatically increased after CAP treatment (Figure 3a) in a dose-dependent manner. This indicates that CAP-induced cell cycle block is probably a result of ROS-induced damage through inserting the Guanine bases of DNA.

At the same time, this study also checked the potential influence of CAP treatment on proteins and lipids. For this purpose, we conducted protein carbonyl analysis, a marker of early oxidative damage in proteins [32]. This was done by detecting the stable dinitrophenylhydrazine (DNP) hydrazone adduct formed from the reaction between 2,4-dinitrophenylhydrazine (DNPH) and protein carbonyl group [19]. Figure 3b shows that the carbonyl content of proteins increases with the increase in plasma treatment time. For example, the carbonyl content of proteins significantly increased after CAP treatment, with the growth of 0.06 μmol/mg prot, 0.12 μmol/mg prot, and 0.23 μmol/mg prot for 1 min, 3 min, and 5 min, respectively. Since protein carbonylation can lead to protein degradation or cross-linking, our data demonstrated that CAP treatment could cause protein denaturation, leading to reduced biological activity. Notably, carbonyl assay only shows the overall status of proteins; the specific proteins that were affected by CAP treatment still need to be further investigated. 

The effect of CAP treatment on lipids was studied by analyzing malondialdehyde (MDA), which is an important indicator of lipid peroxidation damage and is one of the causes of cellular damage [33]. As shown in Figure 3c, with the increase in plasma treatment time, the level of MDA in the tested esophageal cancer cells displayed a steady increase. Compared with the untreated group (0 min treatment), the MDA content of the 5 min treatment group significantly increased from 0.28 nmol/mg prot to 0.63 nmol/mg prot, indicating that CAP-mediated cell death is associated with lipid oxidation damage.

In our preliminary experiments, we conducted comparative studies on both cell lines (Ec9709 and Eca109) and observed minimal differences between them. Moreover, the identical mechanisms of action of 8-OHdG, protein carbonyl content, and MDA have been confirmed by different studies [34,35]. Therefore, only the relevant results of the Ec9706 cell line were presented in the article.

### 2.4. CAP Treatment Induced Heightened Genes Expression of Redox and Glutamine Pathways

**Figure 4:**
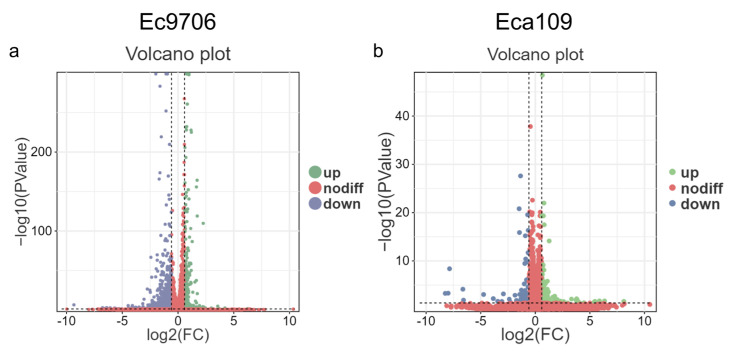
Volcano diagram analysis of differentially expressed genes in esophageal cancer cells after CAP treatment. (**a**,**b**) Volcano plot analysis of differentially expressed genes in Ec9706 and Eca109 cells, *p* < 0.05. Note: blue represents significantly upregulated genes, green represents significantly down-regulated genes, and red represents no difference.

In order to elucidate the mechanism of ROS-associated anticancer effects after CAP treatment, we performed transcriptome sequencing analysis on Ec9706 and Eca109 esophageal cancer cell models treated with CAP for 0 min (control) and 3 min. As shown in Figure 4, after CAP treatment, the expression of a large number of mRNAs displayed either upregulation or down-regulation in both esophageal cancer cell lines (*p* < 0.05).

**Figure 5:**
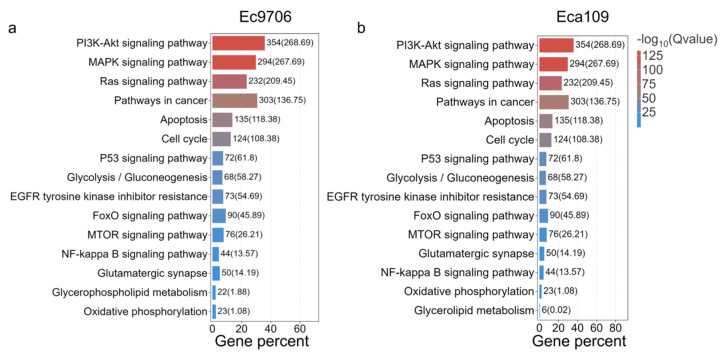
KEGG enrichment analysis of mRNA in esophageal cancer cells after CAP treatment. (**a**,**b**) Analysis of KEGG after Ec9706 and Eca109 cells’ sequencing.

Given the important role of pathway analysis in understanding the biological functions of individual genes, we set out to identify the detailed pathways that are involved in CAP-based anticancer effects through an enrichment analysis [36]. This is performed by using the KEGG (Kyoto Encyclopedia of Genes and Genomes) database by linking genomic information with higher-order functional information [37]. As shown in Figure 5, after applying a series of hypergeometric tests, pathways that are significantly enriched in the target gene sets compared with the background gene sets are identified to be closely related to CAP-based cancer therapy. These include the apoptosis-related PI3K-Akt signaling pathway [38], p53 signaling pathway [39], MAPK signaling pathway [40], and K-Ras [41] pathway.

**Figure 6:**
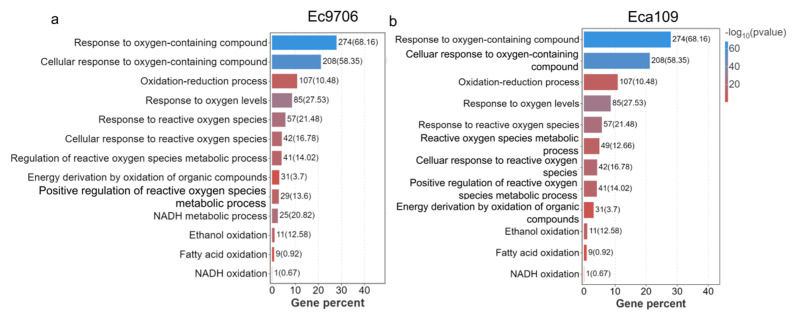
GO analysis of esophageal cancer mRNA after CAP treatment. (**a**,**b**) GO analysis after Ec9706 and Eca109 cells’ sequencing.

Gene Ontology (GO) is a database established by the Gene Ontology Alliance in which differentially expressed genes can be enriched and classified into biological processes, cellular components, and molecular functions [42,43]. To determine the target gene enrichment expression after CAP treatment, we further mapped the target genes of esophageal cancer cells treated with CAP to different terms of the GO database and calculated the number of genes in each term to obtain a list of genes with a certain GO function. After applying hypergeometric testing, we observed significant enrichment in the target genes compared with the entire genome background (Figure 6). Screened all gene sets detected in the sample to obtain genes related to the cellular redox pathway, generated a target gene set, associated the target gene set with the GO database, performed gene enrichment analysis, and obtained GO items with significantly enriched genes. As shown in Figure 6, after CAP treatment, there was a significant enrichment in genes related to the oxidation-reduction process. In addition, genes related to the regulation of the metabolic process of reactive oxygen species also demonstrated enrichment, indicating that CAP treatment may disrupt cellular redox homeostasis and regulate redox metabolism.

**Figure 7:**
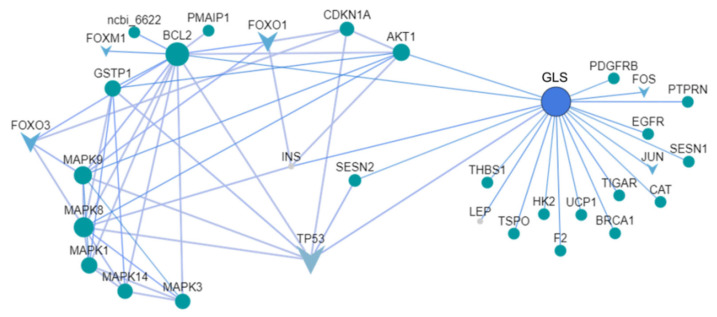
PPI network diagram of esophageal cancer cells after CAP treatment. Apoptosis-related genes are closely related to glutaminase (GLS) in the glutathione precursor glutamine metabolism pathway. GLS: glutaminase, TP53: Tumor protein p53, BCL2: B-cell lymphoma-2.

As the protein–protein interaction network (PPI) can reflect the interactions between proteins (genes), average gene abundance, and connectivity, we analyzed the PPI network diagram of esophageal cancer cell-related proteins (genes) after CAP treatment [44] to elucidate potential pathways associated with the anticancer effects of CAP. As shown in Figure 7, in the PPI network diagram of treated cancer cells, apoptosis-related genes are closely related to glutaminase (GLS) in the glutathione precursor glutamine metabolism pathway, indicating that CAP may control glutathione content by regulating glutamine metabolism, thereby affecting intracellular redox processes and leading to cell apoptosis.

### 2.5. CAP Could Reduce Glutamine Metabolism

**Figure 8:**
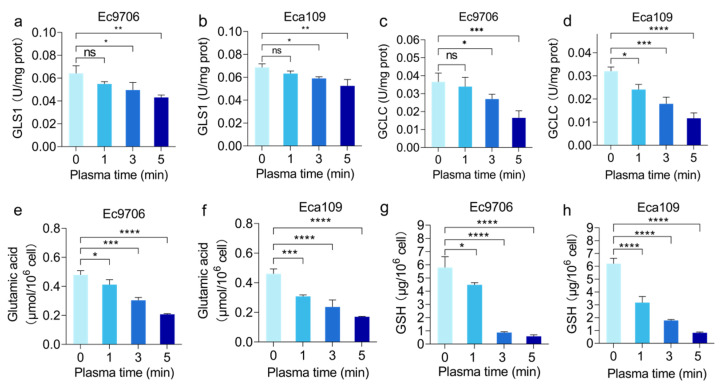
Analysis of changes in the glutamine metabolism pathway in esophageal cancer cells after CAP treatment. (**a**,**b**) Glutaminase 1 enzyme activity in Ec9706 and Eca109 cells. (**c**,**d**) γ-glutamylcysteine ligase enzyme activity in Ec9706 and Eca109 cells. (**e**,**f**) Glutamate content in Ec9706 and Eca109 cells. (**g**,**h**) Glutathione content in Ec9706 and Eca109 cells. (* representing *p* < 0.05; ** representative *p* < 0.01; *** Representing *p* < 0.001; **** representative *p* < 0.0001; ns represents *p* > 0.05).

Given the important role of glutamine, the precursor of the antioxidant glutathione, in promoting oxidative damage and apoptosis of cancer cells in vivo, as shown in the above results, some key enzymes related to glutamine metabolism were analyzed. Glutaminase 1 (GLS 1) is the first and rate-limiting enzyme in catalyzing the breakdown of glutamine by converting glutamine into glutamate [45]. In order to further verify the changes in Glutaminase 1 after CAP treatment, we analyzed the glutaminase 1 enzyme activity in both esophageal cancer cells after treatment. As shown in Figure 8a,b, with the increase of CAP treatment time, the Glutaminase 1 enzyme activity in Ec9706 and Eca109 cells gradually decreased. Among them, the Glutaminase 1 enzyme activity in Ec9706 cells decreased significantly after CAP treatment for 5 min compared with the control group (0 min) (*p* < 0.01), and similarly, the Glutaminase 1 enzyme activity in Eca109 cells also decreased significantly after 5 min CAP treatment (*p* < 0.01). These results indicate that CAP treatment can inhibit Glutaminase 1 enzyme activity in esophageal cancer cells, thereby suppressing glutamine metabolism.

In addition to Glutaminase 1, γ-glutamylcysteine ligase (GCLC) is another rate-limiting enzyme in glutathione synthesis, responsible for catalyzing the first step of glutathione synthesis, which is to link glutamate with cysteine to form γ-glutamylcysteine ligase, thereby regulating the rate of glutathione synthesis [46,47,48]. The results of this study indicate that CAP treatment can significantly reduce intracellular γ-glutamylcysteine ligase enzyme activity. As shown in Figure 8c, the γ-glutamylcysteine ligase enzyme activity in Ec9706 cells significantly decreased after CAP treatment compared with the control group (0 min), especially in cells treated with CAP for 5 min where the γ-glutamylcysteine ligase enzyme activity decreased to about half of the control group. Similar results were collected from the Eca109 esophageal model. As shown in Figure 8d, compared with the control group (0 min), the γ-glutamylcysteine ligase enzyme activity in the CAP treatment group significantly decreased. After 5 min of CAP treatment, the γ-glutamylcysteine ligase enzyme activity decreased to about one-third of the control group. These results indicate that CAP can significantly reduce the activity of γ-glutamylcysteine ligase enzyme in esophageal cancer cells, thereby inhibiting the production of glutathione pathway through glutamine metabolism.

Glutaminase 1 catalyzes the conversion of glutamine to glutamate. To gain more information about how CAP treatment affected glutamine metabolism, the content of glutamate was tested in two esophageal cancer cells treated with CAP, as shown in Figure 8e,f. With the prolongation of plasma treatment time, the level of glutamate gradually decreased. The intracellular glutamate level of Ec9706 cells significantly decreased compared with the control group after 1 min of CAP treatment (*p* < 0.05), and the glutamate content decreased even more after 3 min of CAP treatment (*p* < 0.001). After 5 min of CAP treatment, the intracellular glutamate content decreased to about 50% of the control group (*p* < 0.0001). Similar results were observed with another esophageal cancer cell line, Eca109. After 1 min, 3 min, and 5 min of CAP treatment, the intracellular glutamate content showed a time-dependent decrease, which was significantly different from that of the control group. Among them, the intracellular glutamate level decreased by 50% after 5 min of CAP treatment. From this, it can be inferred that CAP treatment can regulate the activity of glutaminase, thereby inhibiting the metabolism of glutamine.

Glutathione (GSH) is a product of the glutamine metabolic pathway. This study also detected the changes in intracellular glutathione content after CAP treatment at different times. The results are shown in Figure 8g,h. The glutathione levels in esophageal cancer cells after CAP treatment showed a decreasing trend. The glutathione levels in the 3 min and 5 min sample groups decreased sharply compared with the control group, especially the glutathione level in the 5 min sample group, which was about 1/10 of the control group (*p* < 0.0001). This study also analyzed the changes in intracellular glutathione content in another esophageal cancer cell line, Eca109. After CAP treatment, the intracellular glutathione level in Eca109 cells significantly decreased. The sample group treated with CAP for 1 min had a decrease in intracellular GSH level of half of the control group, while the sample groups treated with CAP for 3 and 5 min had a sharp decrease in intracellular glutathione level. The glutathione level in the 5 min sample group was significantly reduced to about 1/10 of the control group (*p* < 0.0001). Therefore, CAP does indeed reduce intracellular glutathione levels, which in turn affects intracellular ROS clearance.

### 2.6. CAP Treatment Significantly Affects the Expression of Glutamine Metabolism-Related Genes

To further investigate the correlation between glutamine metabolism change and apoptosis after CAP treatment, our study analyzed the gene expression changes related to glutamine metabolism and apoptosis in esophageal cancer cells after plasma treatment using qRT-PCR. c-Myc can induce the transcription of glutamine transporter SLC1A5 and promote the expression of glutaminase gene [49]. The intermediate product of glutamine metabolism, glutamate, can promote the synthesis of glutathione through the γ-glutamylcysteine ligase enzyme. In addition, the uptake of glutamine is negatively regulated by the Rb gene family [50]. K-Ras can upregulate the expression of glutamine transporter SLC1A5, and NRF2 upregulates the expression of γ-glutamylcysteine ligase (GCLC) and glutathione synthase (GSS) [51], thereby promoting glutathione synthesis. Therefore, this article detected the above-mentioned gene expression changes in two esophageal cancer cell lines after CAP treatment.

**Figure 9:**
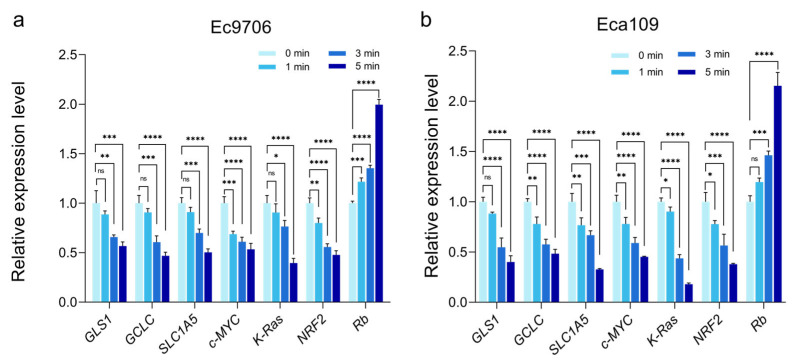
Expression of glutamine metabolism-related genes after CAP treatment. (**a**,**b**) Expression of glutamine metabolism-related genes in Ec9706 and Eca109 cells. (* representing *p* < 0.05; ** representative *p* < 0.01; *** Representing *p* < 0.001; **** representative *p* < 0.0001; ns represents *p* > 0.05).

According to Figure 9, the gene expression of glutamine transporter SLC1A5 in both esophageal cancer cells treated with CAP showed a decreasing trend compared with the untreated cell group (0 min). Analysis of the inhibitory gene Rb in the glutamine transport system revealed an increasing trend in Rb gene expression after CAP treatment. After 5 min of CAP treatment, the expression level of the Rb gene was about twice that of the untreated group (0 min), indicating that CAP treatment inhibited the expression of glutamine transporter SLC1A5, thereby inhibiting cell uptake of glutamine and reducing intracellular glutamine sources. In addition, the expression of glutaminase 1 gene in the two cell lines in Figure 9 decreased significantly with treatment time compared with the control group (0 min), indicating that CAP inhibited the expression of glutaminase 1 in cells. Similarly, the expression of the γ-glutamylcysteine ligase gene also showed a downward trend compared with the control group, indicating that CAP inhibited the expression of the γ-glutamylcysteine ligase gene. This suggests that CAP can indeed inhibit the metabolic pathway of glutamine to produce glutathione. c-Myc can induce transcription of SLC1A5 and promote the expression of glutaminase 1 and γ-glutamylcysteine ligase [52]. CAP treatment inhibited the expression of the c-Myc gene, further indicating that CAP can indeed inhibit the metabolic pathway of glutamine to produce glutathione. This study also detected the relative expression of K-Ras that can upregulate the glutamine transporter SLC1A5. The results showed that the CAP treatment group could significantly inhibit the expression of K-Ras. The relative expression levels of the two esophageal cancer cells in the CAP treatment group for 5 min were less than 50% of the control group, indicating that CAP may inhibit the expression of glutamine transporter SLC1A5 in esophageal cancer cells by suppressing the expression of related genes such as K-Ras. NRF2 can upregulate the expression of γ-glutamylcysteine ligase and glutathione synthase (GSS) in the glutathione pathway [53]. qRT-PCR results showed that CAP treatment could inhibit the expression of the NRF2 gene in esophageal cancer, indicating that CAP may suppress the expression of γ-glutamylcysteine ligase and glutathione synthase (GSS) by inhibiting the expression of NRF2 gene, thereby inhibiting the glutamine metabolism and glutathione pathway in esophageal cancer cells.

### 2.7. CAP Treatment Significantly Affects the Expression of Cell Cycle and Apoptosis-Related Genes

**Figure 10:**
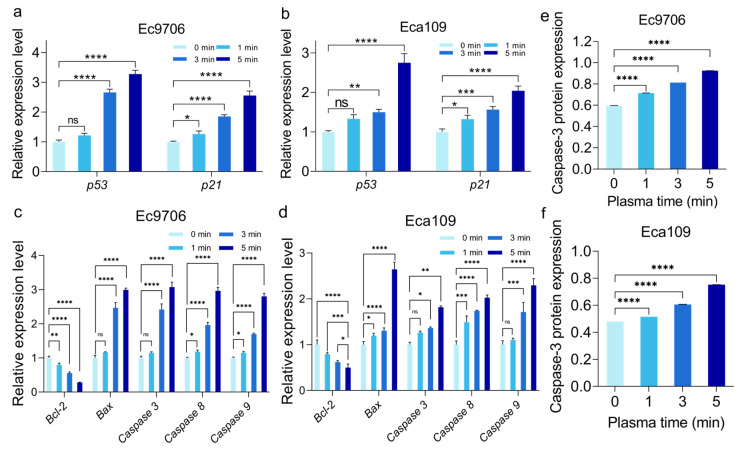
Analysis of cell cycle and apoptosis-related genes and protein after CAP treatment. (**a**,**b**) Ec9706 and Eca109 cell cycle-related gene expression. (**c**,**d**) Apoptosis-related gene expression in Ec9706 and Eca109 cells. (**e**,**f**) Relative expression of caspase-3 protein in Ec9706 and Eca109 cells. (* representing *p* < 0.05; ** representative *p* < 0.01; *** Representing *p* < 0.001; **** representative *p* < 0.0001; ns represents *p* > 0.05).

The decrease in glutathione production affects the clearance ability of ROS, leading to cell apoptosis and cycle arrest [54]. Therefore, this study also analyzed the expression of genes related to cell cycle arrest and apoptosis in esophageal cancer cells after CAP treatment. p53 mainly activates the cyclin-dependent protein kinase inhibitor p21 through transcription to prevent cells from entering the S phase [55]. p21 can prevent the activation of the cyclin B-CDK1, thereby blocking the cell cycle in the G2/M phase [56]. As shown in Figure 10a,b, the expression of the p53 gene in esophageal cancer cells increased after CAP treatment. After 5 min of treatment, the expression level of the p53 gene in Ec9706 cells was about 3 times that of the control group and showed significant differences (*p* < 0.0001), while the expression level of the p53 gene in Eca109 cells was about 2.5 times that of the control group (0 min) and showed significant differences (*p* < 0.0001). After CAP treatment, the expression of the p21 gene in esophageal cancer cells also significantly increased. Among them, the expression level of the p21 gene in Ec9706 cells after 5 min of CAP treatment increased by more than twice compared with the control group (0 min) (*p* < 0.0001), and the expression level of the p21 gene in Eca109 cells also significantly increased compared with the control group (0 min) (*p* < 0.0001). It can be inferred that CAP treatment can upregulate the expression of p53 and p21 genes in esophageal cancer cells, block the progression of the cell cycle, and thus inhibit cell proliferation.

At the same time, this study analyzed the expression changes of apoptosis-related genes after CAP treatment. The increase in intracellular ROS is the main trigger of cell apoptosis [57], and the Bcl-2 protein in the ROS-related apoptosis pathway can inhibit cell apoptosis, while Bax and caspases can induce cell apoptosis [58]. As shown in Figure 10c,d, the expression of the Bcl-2 gene that inhibits apoptosis in both esophageal cancer cells significantly decreased (*p* < 0.05) after CAP treatment, while the expression of Bax, caspase-3, caspase-8, and caspase-9 genes that promote apoptosis significantly increased (*p* < 0.05), and showed a time-dependent effect. Caspase-3 is the central effector caspase in both the intrinsic (mitochondrial) and extrinsic (death receptor) apoptotic pathways. Its activation is considered a definitive indicator of apoptosis commitment, regardless of the upstream initiating signals (caspase-8 or caspase-9) [59]. In addition, as shown in Figure 10e,f, the expression level of the caspase 3 protein, which promotes cell apoptosis, also significantly increased. This indicates that CAP treatment induces cell apoptosis by upregulating the expression of pro-apoptotic genes and down-regulating the expression of anti-apoptotic genes due to the inability to clear ROS.

## 3. Discussion

Cold atmospheric plasma (CAP), as a novel molecular activation method, has attracted the attention of researchers across various areas of biomedical fields, including microbial sterilization, dermatitis, wound healing, and cancer treatment [60,61,62,63,64,65,66,67]. 

Previous studies have found that CAP could specifically induce apoptosis in many types of cancers, such as breast cancer [68] and rectal cancer [69,70,71,72,73,74], displaying great promise for the development of a novel anticancer approach [75]. Further investigation revealed that this cancer-specific apoptosis induction involved two pathways, either altering the energy supply of cancer cells or blocking the cell cycle. Moreover, both pathways rely on oxidative damage caused by the reactive oxygen species of CAP [57]. For example, it was found that the ROS/caspase-9/caspase-3 apoptotic pathway was activated by CAP exposure, leading to Gasdermin E (GSDME) lysis and cancer cell pyroptosis [76]. Our findings are highly consistent with these observations. As shown in the KEGG and GO enrichment analysis (Figure 5 and Figure 6), there was a significant enrichment of genes related to redox processes in esophageal cancer cells after CAP treatment, as well as enrichment of apoptosis-related signaling pathways such as p53 and oncogenes K-Ras. 

In one of our recent publications, we observed significantly reduced glutamine levels after CAP treatment in different cancer cell models. As known, glutamine is a precursor for the synthesis of glutathione; reduced glutamine metabolism suggests a reduction in glutathione production. As glutathione has been proven to serve as an important antioxidant in cells to reduce the cytotoxicity caused by ROS, in all likelihood, the observed ROS elevation after CAP stimulation could be a result of the reduced glutamine–glutathione transition. Investigating the detailed mechanism involved in this transition provides a foundation to further improve the efficacy of existing CAP treatment. 

**Figure 11:**
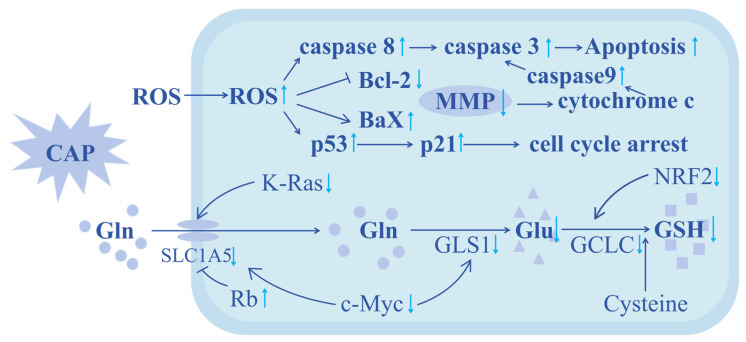
A diagram showing the potential mechanisms of CAP-mediated cancer cell apoptosis. In summary, through exploring the changes in the enzymes and genes involved in glutamine metabolism, some key factors involved in CAP-based cancer inhibition were identified. ↑ indicates an increase in relative expression level, and ↓ indicates a decrease in relative expression level.

There are multiple pathways involved in glutamine–glutathione transition; these include SLC1A5, glutaminase 1, γ-glutamylcysteine ligase, and glutamate acid (Figure 9 and Figure 10). As glutaminase 1 and γ-glutamylcysteine ligase are both speed-limiting enzymes in the glutamine–glutathione transition, they are systematically investigated in this study. According to our data, CAP treatment is closely related to reduced glutaminase 1 enzyme expression, leading to reduced glutamine metabolism. As glutamine serves as a source for glutathione synthesis, a reduction in cellular glutathione levels is expected (as shown in Figure 11), which likely contributes to CAP-induced cancer cell death. Indeed, cancer cells typically express a high level of antioxidants, with glutathione being one of the most important ones. Research has shown that the deficiency of glutathione can disrupt the redox homeostasis of cells, leading to the accumulation of ROS and ultimately causing cell damage or even death [77]. 

Based on this result, we further investigated the key genes in CAP-mediated anticancer effects. After CAP treatment, we found that in addition to glutaminase 1, the cellular level of another glutamine metabolism promotor, γ-glutamylcysteine ligase, also showed a significant decrease, suggesting that a synergistic glutamine-reducing mechanism exists after CAP treatment. This double-layered glutamine-reducing mechanism, we found, could be further improved by upregulating genes that inhibit glutamine metabolism (e.g., Rb) or down-regulating genes that promote glutamine metabolism (e.g., SLC1A5, GLS1, GCLC, K-Ras, c-Myc, NRF2) (Figure 10). As glutathione is an intracellular antioxidant that can reduce ROS levels to protect cells, the decrease in glutathione leads to compromised ROS clearance and induces cancer cell apoptosis, manifested by significant cell cycle arrest, as shown in our gene expression analysis (Figure 10).

Our findings provide a foundation for the development of novel CAP-mediated anticancer treatments. For example, by combining applying novel macrocyclic inhibitors targeting glutaminase 1 allosteric binding site [78], γ-glutamylcysteine ligase inhibitor buthionine sulfoximine (BSO) [25], the anticancer efficacy of CAP treatment could be further improved.

In recent years, the targeting of tumor metabolism has emerged as a pivotal strategy in cancer therapeutics. Glutaminolysis, functioning as a central metabolic pathway that sustains both energy production and redox homeostasis in tumor cells, has positioned its key enzyme glutaminase (GLS) as a promising therapeutic target. Clinical evidence demonstrates that the GLS inhibitor CB-839 shows significant efficacy against glutamine-dependent tumors [79], particularly those with MYC amplification or KRAS mutations. Mechanistically, the combination of GLS inhibitors with NRF2 pathway inhibitors (e.g., ML385) induces synergistic ROS accumulation through dual blockade of antioxidant defenses [80]. Importantly, cold atmospheric plasma (CAP) exhibits complementary mechanisms with metabolic inhibitors, collectively overcoming the oxidative stress tolerance threshold of malignant cells and substantially enhancing therapeutic efficacy. This will be explored in our subsequent investigations.

Although cold atmospheric plasma (CAP) has demonstrated significant potential in cancer therapy, its clinical application still faces substantial challenges due to the complexity of its mechanisms, difficulties in standardizing dose-response relationships, and limited penetration depth, which restricts its use to surface or superficial tumors while proving inadequate for deep-seated solid tumors. Additionally, this research was performed only in cell lines and needs to be tested in animal models. Due to space limitations, we focused our testing on the critical caspase-3. Further research will require complementary measurement data for caspase-9. Thus, extensive research is still required to advance CAP to the level of clinical therapeutics. We remain committed to further investigating and optimizing CAP-based treatment protocols to enhance therapeutic efficacy.

## 4. Method and Material

### 4.1. Cell Culture

Ec9706 cells were derived from the School of Medicine of ZhengZhou University, cultured in DMEM medium containing 10% fetal bovine serum (FBS); Eca109 cells were derived from Professor Huang Qing’s group at Hefei Institute of Physical Sciences, Chinese Academy of Sciences, cultured in RPMI 1640 medium containing 10% fetal bovine serum (FBS). Both types of cells were cultured in a humidified incubator containing 5% carbon dioxide at 37 °C.

### 4.2. Plasma Device 

**Figure 12:**
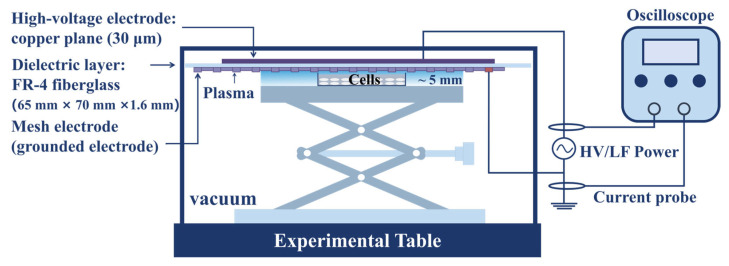
A schematic diagram of the experimental setup. The plasma was used with a power of 7.5 W, a pulse frequency of 7 kHz, and an airflow rate controlled at around 1 L/min. The cells are at a distance of 1 mm from the discharge plate. The grids of the grounding electrode are all hexagonal (the side length of the regular hexagonal grid is 4.62 mm), and the entire discharge area is 65 mm× 70 mm.

The cold atmospheric plasma device (Figure 12) used in this study consists of a plasma generator, an acrylic glass chamber, an air pump (Outstanding, OTS-800, Hangzhou, China), and a dielectric barrier surface discharge plasma device, which includes an oscilloscope (Teledyne WaveSurfer 3000, Hangzhou, China). In this study, the plasma was used with a power of 7.5 W, a pulse frequency of 7 kHz, and an airflow rate controlled at around 1 L/min. The cells are at a distance of 1 mm from the discharge plate. We used a 30 µm thick copper flat electrode (high voltage) and a hexagonal mesh electrode as the electrodes of the device, with a 1.6 mm FR-4 glass fiber dielectric substrate serving as the dielectric layer. The grids of the grounding electrode are all hexagonal (the side length of the regular hexagonal grid is 4.62 mm), and the entire discharge area is 65 mm × 70 mm [81]. Place approximately 3 mL of cell suspension in a Φ 35 mm culture dish container, and the surface was about 1 mm away from the electrode plate. The cells were divided into four groups and treated for 0 min, 1 min, 3 min, and 5 min, respectively. The treated cells were further cultured in a 5% carbon dioxide incubator at 37 °C.

### 4.3. Cell Viability Analysis 

Cells were treated with CAP for different times (0, 1, 3, 5 min). Cells were then seeded into a 96-well plate (3000 cells/well/180 μL medium) and cultured for 24 h. After incubation, 20 μL of 5 mg/mL MTT solution (Solarbio, Beijing, China) was added into the cell culture and incubated for an additional 4 h. Cell viability was calculated by measuring absorbance at 490 nm using a microplate reader (Varioskan LUX, Thermo Scientific, Waltham, MA, USA). This analysis was repeated three times to evaluate the consistency of the results. The formula for calculating cell viability is the following:

Cell viability = (ODcontrol/ODtreat) × 100%

### 4.4. Intracellular ROS Analysis 

After indicated CAP treatments, cells were collected (5 × 10^6^), followed by adding 1 mL of 2′, 7′-dichlorodihydrofluorescein diacetate (DCFH-DA) with a final concentration of 10 µM. Cells were then incubated at 37 °C for 20 min. After incubation, the cells were washed three times with 1 mL of serum-free cell culture medium via centrifugation at 1000× *g* for 5 min. Add 1 mL of DMEM medium (containing 10% FBS) and resuspend the cells. Fluorescence intensity was measured using a NovoCyte3 130 flow cytometer (Agilent, PAMF, Palo Alto, CA, USA) at 488 nm excitation and 525 nm emission wavelength. The data were analyzed using FlowJo 10.6.2 and GraphPad 8.0.2.

### 4.5. Cell Cycle Analysis

The DNA staining solution was prepared by adding 20 μL of Triton X-100, 400 μL of 1 mg/mL PI, and 4 mg of DNase-free RNase A to PBS (final volume adjusted to 20 mL). Cells were cultured in T25 flasks for 48 h following CAP treatment and then collected in a 1.5 mL EP tube at a concentration of 106 cells per tube. After centrifugation at 1000× *g* for 5 min, the supernatant was discarded. The cells were then resuspended and washed three times with pre-cooled PBS at 4 °C. After the final wash; the cells were resuspended in 300 μL of PBS, followed by the addition of 700 μL of pre-cooled anhydrous ethanol, which was added dropwise. The cells were then fixed at 4 °C for 24 h. After fixation, the cells were centrifuged at 1000× *g* for 5 min. After a single wash with PBS to remove residual ethanol, 1 mL of DNA staining solution was added, and the sample was incubated in the dark at 37 °C for 15 min. The PI red fluorescence was then detected using the NovoCyte3 130 flow cytometer (Agilent, USA) through the PE channel. The results were analyzed using FlowJo 10.6.2 software.

### 4.6. Apoptosis Assay

Esophageal cancer cells (Ec9706 and Eca109), which were treated with CAP, were cultured in a 37 °C incubator with 5% CO_2_ for 24 h. After incubation, the cells were digested using EDTA-free trypsin (Solerbao, Beijing, China) and then resuspended in PBS. After centrifuging at 1000 g for 5 min, the supernatant was discarded, and the cells were then collected (5 × 10^5^). The level of apoptosis was then detected following CAP treatment using the Annexin V-FITC/PI apoptosis assay kit (Boster, Wuhan, China). Then, 5 μL Annexin V-FITC and 5 μL PI were added into this system with 500 μL binding buffer. After incubating cells at room temperature in the dark for 15 min, apoptosis was detected with a NovoCyte3 130 flow cytometer (Agilent, USA). The data were analyzed using FlowJo 10.6.2 and GraphPad 8.0.2.

### 4.7. DNA Extraction

After CAP treatment, the DNA of Ec9706 and Eca109 cells was extracted using a DNA extraction kit (Kangwei Century, Taizhou, China). The concentration of DNA was adjusted to 5 μg. Subsequently, 5 U of DNase I (Kangwei Century, Taizhou, China), 0.005 U of snake venom phosphodiesterase (Sigma Aldrich, Saint Louis, MO, USA), and 100 µM methanesulfonic acid deferoxamine (Ron, Shanghai, China) were added to the cell culture. After incubation at 37 °C for 1 h, 2.0 U of calf intestinal phosphatase (New England Biolabs, Ipswich, MA, USA) was added. The enzymes were then removed using a 3 kDa ultrafiltration tube after reacting at 37 °C for 30 min and incubating at 80 °C for 2 min. Stored the DNA samples at 4 °C.

### 4.8. 8-OHdG Analysis

UPLC-MS (Acquity UPLC I-Class PLUS, Waters, Milford, MA, USA) was used for analysis. The separation was performed on an ACQUITY UPLC BEH C18 column (2.1 × 50 mm, 1.7 µm) with a temperature of 35 °C. The injection volume was 10 μL, and the mobile phase consisted of (A) 10 mM ammonium formate (NH_4_HCO_2_, Sigma Aldrich, USA) and (B) acetonitrile (Fisher, Storrs, CT, USA), with a flow rate of 0.3 mL/min. Gradient elution was employed, followed by ESI ionization and MRM scanning in positive ion mode. 

### 4.9. dG Detection

ACQUITY UPLC H-Class system (Waters, USA) was used with a SpursirTM C18 chromatography column (150 × 4.6 mm, 5 µm, Waters). The column temperature was set at 35 °C, and the injection volume was 10 µL. The mobile phase consisted of A: 10 mM ammonium formate (Sigma Aldrich, USA) and B: acetonitrile (Fisher, USA), with a flow rate of 0.3 mL/min. 

After performing gradient elution, standard curves for 8-OHdG and dG were constructed using standard samples. The content of 8-OHdG and dG were then calculated based on these standard curves. The relative content of 8-OHdG is represented by the results of 8-OHdG/10^5^ dG.

### 4.10. Protein Carbonyl Analysis

Cells were collected after CAP treatment (5 × 10^6^), then sonicated in an ice bath under the conditions of 300 W power, 3 s ultrasound, 7 s interval, and a total time of 3 min after adding 1 mL of extraction solution in the cell culture. Centrifuged at 10,000× *g* and 4 °C for 10 min, then placed supernatant on ice. Following operation with a protein carbonyl content detection kit (Bioss, Beijing, China), the OD value was measured at a wavelength of 370 nm using a microplate reader (Varioskan LUX, Thermo Scientific, Waltham, MA, USA). The protein carbonyl content was then calculated according to the formula provided in the kit’s instructions.

### 4.11. Analysis of Malondialdehyde 

After indicated CAP treatments, 1 mL of extraction solution was added to the reaction system. Cells were then sonicated using a cell disruptor under the conditions of 20% power, 3 s ultrasound, 10 s intervals, and repeated for 30 cycles. The supernatant was collected and used for detecting malondialdehyde (MDA) content with an MDA detection kit (Solarbio, China). The absorbance of the sample was measured at 450 nm, 532 nm, and 600 nm using a UH4150 UV-Visible Spectrophotometer (Hitachi, Tokyo, Japan). The MDA content was then calculated according to the formula provided in the kit’s instruction manual.

### 4.12. Cell Transcriptome Sequencing Analysis

Total RNA was extracted from esophageal cancer cells that had been treated with CAP after further cultured for 24 h, and mRNA was enriched from it. After enriching eukaryotic mRNA with polyA tails using magnetic beads with Oligo (dT), the mRNA was cleaved using a buffer. Using fragmented mRNA as a template and random oligonucleotides as primers, the first strand of cDNA was synthesized in the M-MuLV reverse transcriptase system. Subsequently, the RNA strand was degraded using RNaseH, and the second strand of cDNA was synthesized from dNTPs in the DNA polymerase I system. After purification, the double-stranded cDNA was subjected to terminal repair, followed by the addition of an A tail and the connection of sequencing adapters. Approximately 200 bp of cDNA was screened using AMPure XP beads for PCR amplification, and the PCR product was purified using AMPure XP beads again to obtain a library. mRNA sequencing was performed by Guangzhou GENEDENOVO Co., Ltd. (Guangzhou, China).

### 4.13. Glutaminase 1 Analysis

The cells (104) were collected after CAP treatment. After continued cultured for 24 h, 1 mL of extraction solution was added. The cells were then sonicated on ice to break them apart, with the power at 300 W. Then, they were sonicated for 3 s and paused for 7 s, repeating this cycle until a total sonication time of 3 min was achieved. After sonication, the mixture was centrifuged at 4 °C and 12,000× *g* for 15 min, and the supernatant was collected and placed on ice for subsequent analysis. The intracellular content of glutaminase was detected using the Glutaminase Assay Kit (Solarbio, China). The solution was prepared according to the kit’s instructions and incubated at room temperature for 30 min. The absorbance was then measured at 630 nm using the UH4150 UV-Visible Spectrophotometer (Hitachi, Tokyo, Japan).

### 4.14. γ-Glutamylcysteine Ligase Analysis

Esophageal cancer cells treated with CAP were collected after further cultured for 24 h and thoroughly mixed with 1 mL of extraction solution. Then, they were sonicated for 3 s and paused for 7 s, repeating this cycle until a total sonication time of 3 min was achieved. After complete cell lysis, the mixture was centrifuged at 8000× *g*, 4 °C for 10 min. The intracellular content of γ-glutamylcysteine ligase was detected using the γ-glutamylcysteine ligase Assay Kit (Solarbio, China). The solution was prepared according to the kit’s instructions and placed on ice. After mixing thoroughly, the container was sealed and incubated in a 45 °C water bath for 10 min. The absorbance was then measured at 630 nm using the UH4150 UV-Visible Spectrophotometer (Hitachi, Tokyo, Japan).

### 4.15. Glutamate Analysis

Esophageal cancer cells treated with CAP were collected after further cultured for 24 h. The cells were centrifuged at 1000× *g* for 5 min and discarded the supernatant. Cells were then sonicated with the power at 200 W, which sonicated for 3 s and paused for 7 s; this cycle was repeated 30 times after mixing with 1 mL of extraction reagent. After complete cell lysis, the mixture was centrifuged at 1000× *g* and 4 °C for 10 min. Detected changes in glutamate content with a Glutamate Assay Kit (Solarbio, China), and the solution was then prepared according to the kit’s instructions. The absorbance was measured at 340 nm using the UH4150 UV-Visible Spectrophotometer (Hitachi, Tokyo, Japan).

### 4.16. Glutathione Analysis

Esophageal cancer cells treated with CAP were collected after being cultured further for 24 h. The cells were centrifuged at 600× *g* for 10 min with 1 mL PBS. Cells were then sonicated with the power at 200 W, which sonicated for 3 s and paused for 10 s; this cycle was repeated 30 times after mixing with 1 mL of extraction reagent. After sonication, the mixture was centrifuged at 4 °C and 8000× *g* for 10 min, and the supernatant was collected and placed on ice for subsequent analysis. The intracellular content of glutathione was detected using the Glutathione Assay Kit (Solarbio, China). The solution was prepared according to the kit’s instruction and placed on ice, and the absorbance was then measured at 412 nm after incubating at room temperature for 2 min using the UH4150 UV-Visible Spectrophotometer (Hitachi, Tokyo, Japan).

### 4.17. Relative Content Detection of Caspase-3

Esophageal cancer cells treated with CAP were collected in a culture dish (Φ 35 mm) in a humidified incubator containing 5% carbon dioxide at 37 °C for 8 h. After being centrifuged in a centrifuge tube at 2000× *g* for 5 min, the cells were then planted in a 96-well plate and incubated for 1.5 h in the dark using a Caspase-3 Detection Kit (Beyotime, Shanghai, China). The relative content of caspase-3 was then measured using a microplate reader (Varioskan LUX, Thermo Scientific, Waltham, MA, USA).

### 4.18. qRT-PCR Analysis

After CAP treatment, the cells were further cultured (Φ 60 mm) for 24 h and then lysed with 1 mL of TRIzol (Thermo Fisher, USA). Subsequently, 200 μL of chloroform was added, and the mixture was thoroughly mixed well. After incubating at room temperature for 5 min. The supernatant was then centrifuged for 15 min, and 500 μL of isopropanol was added. The cells were left to stand at room temperature for 10 min. After centrifugation, the supernatant was discarded, and 1 mL of 75% ethanol was added for further centrifugation. The supernatant was dried at room temperature on a clean bench for 10 min to evaporate the ethanol. Finally, 30–50 μL of RNase-free water was added to dissolve the precipitate, which is the RNA solution. The HiFiScript cDNA Synthesis Kit (Kangwei Century, China) was utilized for the reverse transcription of RNA. The reaction system and conditions were referred to in the kit instructions. Real-time fluorescence quantitative PCR was conducted using the Mastercycler ep realplex (Eppendorf, Hamburg, Germany), with β-actin as the internal reference. The specific primer sequences for each gene were sourced from NCBI Primer Blast, and the primer sequences are shown in the supporting materials (Table S1).

### 4.19. Statistical Analysis

All experiments in this study were repeated three times or more. Single-factor analysis of variance (ANOVA) was performed using GraphPad Prism 8 software to determine differences between different treatments, and the Turkey test was used. The Turkey test is one of the multiple comparison methods used when multiple treatment groups have equal sample sizes. (* representing *p* < 0.05; ** representative *p* < 0.01; *** Representing *p* < 0.001; **** representative *p* < 0.0001; ns represents *p* > 0.05).

## 5. Conclusions

This study provides evidence that CAP has a regulatory effect on glutamine metabolism, leading to reduced glutathione levels. We found that there are a large number of genes enriched in redox-related pathways, as well as cycle arrest and apoptosis-related pathways after CAP treatment. Specifically, genes promoting glutamine–glutathione transition, including glutaminase 1, SLC1A5, K-Ras, γ-glutamylcysteine ligase, c-Myc, and NRF2, showed reduced expression, leading to excessive cellular ROS level and enhanced cancer cell apoptosis. This study not only improves our understanding of the mechanism behind CAP-mediated anticancer effects but also provides potential targets to further improve the efficacy of CAP-based cancer treatment. 

## Figures and Tables

**Table 1:** Comparative mechanisms of CAP vs. radiotherapy (preclinical evidence).

	Targeting Mode	Side Effects	Mechanism	Equipment/Application
**CAP**	Specifically targeting cancer cells [13]	Low side effects, can be used in sensitive areas (e.g., neck, brain) [14]	Activate apoptosis-relevant enzymes (i.e., mitogen-activated protein kinase, caspases) [15,16]	Low cost; Low/safe temperature in the treatment area; No specific biosafety facility required.
**Radiotherapy**	Nonspecific cytotoxicity	High	Inducing apoptosis mainly through DNA double-strand breakage [17]	Higher cost Higher local temperature; Need a protective facility.

Note: This table summarizes in vitro and animal study data. Clinical efficacy comparisons require future randomized trials.

## Data Availability

The original contributions presented in this study are included in the article. Further inquiries can be directed to the corresponding authors.

## Supplementary Materials

The following supporting information can be downloaded at https://www.mdpi.com/article/10.3390/molecules30132808/s1, Table S1: Primer sequences of target genes for Real-time fluorescence quantitative PCR.
