# Supplementary material for: The Cold Atmospheric Plasma Inhibits Cancer Proliferation Through Reducing Glutathione Synthesis
Source: Molecules. 2025 Jun 30;30(13):2808. doi: 10.3390/molecules30132808 (PMC12251158; doi:10.3390/molecules30132808)
Supplement: Supplementary file 1 [file molecules-30-02808-s001.zip › molecules-3639696-supplementary.pdf]

Supporting materials

Table S1. Primer sequences of target genes for Real-time fluorescence quantitative PCR

Primer name		Primer sequences（5’-3’）
β-actin	F-primer	CATGTACGTTGCTATCCAGGC
β-actin	R-primer	CTCCTTAATGTCACGCACGAT
Bax	F-primer	CCCGAGAGGTCTTTTTCCGAG
Bax	R-primer	CCAGCCCATGATGGTTCTGAT
Bcl-2	F-primer	GGTGGGGTCATGTGTGTGG
Bcl-2	R-primer	CGGTTCAGGTACTCAGTCATCC
Caspase 3	F-primer	CATGGAAGCGAATCAATGGACT
Caspase 3	R-primer	CTGTACCAGACCGAGATGTCA
Caspase 8	F-primer	AGAGTCTGTGCCCAAATCAAC
Caspase 8	R-primer	GCTGCTTCTCTCTTTGCTGAA
caspase-9	F-primer	CTCAGACCAGAGATTCGCAAAC
caspase-9	R-primer	GCATTTCCCCTCAAACTCTCAA
GCLC	F-primer	GGAGGAAACCAAGCGCCAT
GCLC	R-primer	CTTGACGGCGTGGTAGATGT
GLS1	F-primer	AGGGTCTGTTACCTAGCTTGG
GLS1	R-primer	ACGTTCGCAATCCTGTAGATTT
K-Ras	F-primer	ACAGAGAGTGGAGGATGCTTT
K-Ras	R-primer	TTTCACACAGCCAGGAGTCTT
c-MYC	F-primer	GGCTCCTGGCAAAAGGTCA
c-MYC	R-primer	CTGCGTAGTTGTGCTGATGT
NRF2	F-primer	TCCAGTCAGAAACCAGTGGAT
NRF2	R-primer	GAATGTCTGCGCCAAAAGCTG
p21	F-primer	TGTCCGTCAGAACCCATGC
p21	R-primer	AAAGTCGAAGTTCCATCGCTC
Rb	F-primer	CATCACACCCCAGAGAAGCC
Rb	R-primer	AGAGGGCCGTGCGGTTGGCA
SLC1A5	F-primer	GAGCTGCTTATCCGCTTCTTC
SLC1A5	R-primer	GGGGCGTACCACATGATCC
p53	F-primer	CAGCACATGACGGAGGTTGT
p53	R-primer	TCATCCAAATACTCCACACGC
